# Shrubland primary production and soil respiration diverge along European climate gradient

**DOI:** 10.1038/srep43952

**Published:** 2017-03-03

**Authors:** Sabine Reinsch, Eva Koller, Alwyn Sowerby, Giovanbattista de Dato, Marc Estiarte, Gabriele Guidolotti, Edit Kovács-Láng, György Kröel-Dulay, Eszter Lellei-Kovács, Klaus S. Larsen, Dario Liberati, Josep Peñuelas, Johannes Ransijn, David A. Robinson, Inger K. Schmidt, Andrew R. Smith, Albert Tietema, Jeffrey S. Dukes, Claus Beier, Bridget A. Emmett

**Affiliations:** 1 Centre for Ecology & Hydrology, Environment Centre Wales, Deiniol Rd, Bangor, Gwynedd, LL57 2UW, United Kingdom; 2 School of Environment, Natural Resources and Geography, Bangor University, Bangor, Gwynedd, LL57 2UW, United Kingdom; 3 Council for Agricultural Research and Economics - Forestry Research Centre (CREA-SEL), Viale Santa Margherita, 80 - 52100 Arezzo (AR), Italy; 4 Department for Innovation in Biological, Agro-food and Forest systems (DIBAF), University of Tuscia, Viterbo, Italy; 5 SCIC, Global Ecology Unit, CREAF-CSIC_UAB, Cerdanyola del Vallès, Catalonia, E-08193 Spain; 6 CREAF, Cerdanyola del Vallès, Barcelona, Catalonia, E-08193 Spain; 7 Institute of Agro-Environmental & Forest Biology (IBAF), National Research Council (CNR), Porano, TR, Italy; 8 Institute of Ecology and Botany, MTA Centre for Ecological Research, Alkotmány u. 2-4., 2163-Vácrátót, Hungary; 9 Department of Geosciences and Natural Resource Management, University of Copenhagen, Rolighedsvej 23, 1958 Frederiksberg C, Denmark; 10 Institute for Biodiversity and Ecosystem Dynamics, University of Amsterdam, PO Box 94240, 1090 GE Amsterdam, The Netherlands; 11 Department of Forestry and Natural Resources, Purdue University, West Lafayette, IN, 47907, United States of America; 12 Department of Biological Sciences, Purdue University, West Lafayette, IN, 47907, United States of America

## Abstract

Above- and belowground carbon (C) stores of terrestrial ecosystems are vulnerable to environmental change. Ecosystem C balances in response to environmental changes have been quantified at individual sites, but the magnitudes and directions of these responses along environmental gradients remain uncertain. Here we show the responses of ecosystem C to 8–12 years of experimental drought and night-time warming across an aridity gradient spanning seven European shrublands using indices of C assimilation (aboveground net primary production: aNPP) and soil C efflux (soil respiration: Rs). The changes of aNPP and Rs in response to drought indicated that wet systems had an overall risk of increased loss of C but drier systems did not. Warming had no consistent effect on aNPP across the climate gradient, but suppressed Rs more at the drier sites. Our findings suggest that above- and belowground C fluxes can decouple, and provide no evidence of acclimation to environmental change at a decadal timescale. aNPP and Rs especially differed in their sensitivity to drought and warming, with belowground processes being more sensitive to environmental change.

The fifth IPCC report highlighted the link between atmospheric C dioxide (CO_2_) concentration, plants and soil C turnover^1^. However, researchers typically analyse evidence from above^2^,^3^ and belowground^4^,^5^ C stores separately, or use modelling approaches^1^,^6^,^7^, which means that the direct link between above and belowground C remains poorly understood. Conceptually, terrestrial feedbacks induced by environmental change are mediated by aboveground plant processes: the photosynthetic uptake of atmospheric CO_2_ and the storage of C in plant structures. Belowground autotrophic and heterotrophic processes can result in C loss from terrestrial systems and feed back to the atmosphere. Rates of above and belowground C turnover have been linked to soil hydraulic properties^3^,^8^,^9^ but the effect of environmental change on the magnitude of change in above and belowground C pools remains to be identified. Our data offer empirical evidence for the effects of environmental change on above and belowground ecosystem C pools across a range of European shrublands (Table 1, Supplementary Table S1).

We report on the long-term (decadal) responses of related aNPP and Rs measurements to seasonal reductions of 8–49% mean annual precipitation (MAP) and year-round increases of 0.2–0.9 °C air temperature (MAT), and the impact of these responses on C assimilation and soil C efflux. Sites ranged from xeric to mesic (moderate to well-balanced soil moisture) and hydric (seasonally or permanently waterlogged). The common experimental manipulation setup across sites^10^ allowed for direct comparison^11^ of ecosystem functions across precipitation (West to East) and temperature (North to South) gradients in Europe (see Supplementary Fig. S1 online). Here, we examined responses of aNPP and Rs to drought and warming along an aridity gradient, calculated as a modified Gaussen index (MAP/(2*MAT))^12^. The imposed drought and warming conditions^1^ were defined by changes in the Gaussen index (Supplementary Fig. S2), where a decreasing index indicates drier and warmer climates. The responses of aNPP and Rs to drought and warming are expressed as % change from the untreated control plots (Fig. 1) or as change in C (Supplementary Figs S4–S10).

## Results and Discussion

Site-specific temporal responses of aNPP and Rs to drought and warming varied greatly across sites (Supplementary Figs S4–S10). All sites are shrublands, but differ in plant species composition, soil type and climatic conditions (Table 1) that may drive specific responses to environmental change. Despite the variation across sites, our long-term dataset enables us to investigate progressive and cumulative responses of aNPP and Rs to environmental change. To our knowledge, this is the first study where aNPP and Rs are analysed together on a range of shrublands exposed to long-term climate manipulations. We tested whether shrubland aNPP and Rs respond consistently to drought and warming across sites, despite site-specific variation in ecosystem characteristics.

**Figure 1:**
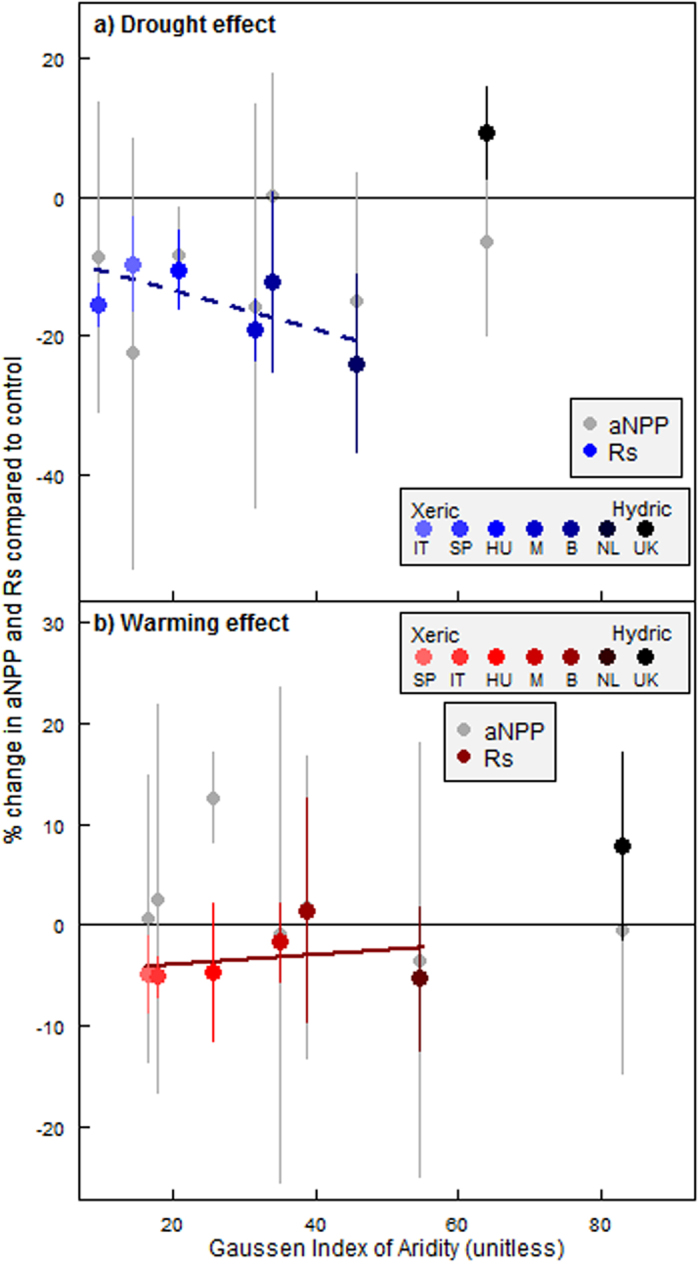
Ecosystem responses to drought and warming across a European aridity gradient. Drought (**a**) and warming (**b**) effects on mean aboveground net primary production (aNPP, grey) and mean soil respiration (Rs, colour gradients) for each experimental site across the Gaussen Index (GI) of Aridity: GI ± standard error (of response means across years). Linear regression was performed across the GI, excluding UK for the Rs response. See text for details. Solid line = significant regression with p < 0.05, dashed line indicates trend with = p = 0.131. IT = Italy, SP = Spain, HU = Hungary, M = Denmark Mols site, B = Denmark Brandbjerg site, NL = The Netherland, UK = United Kingdom.

The effects of precipitation and its legacy effect on aNPP have been explored for arid and sub-humid ecosystems^2^ and, more recently, across shrublands and forests^3^. A positive correlation between aNPP and MAP has been demonstrated and this relationship was maintained in experimentally dried ecosystems^2^,^3^. Here, despite the site-specific interannual variation in aNPP (Supplementary Figs S4–S10, Supplementary Table S2), drought reduced aNPP (*p* = 0.008, Fig. 1a) at all sites compared to the control. We show that the effects of long-term continuous moderate droughts superimposed on natural droughts reduced aNPP across European shrublands. Rs was reduced as a consequence of drought compared to control, leading to a decreased soil C efflux of 10–25% (*p* = 0.001, Fig. 1a) for all but the hydric UK site. At the hydric site, Rs tended to be increased (9%), although not statistically significant (Supplementary Fig. S4). This increase in Rs has been explained by a change in soil structure that led to a permanent reduction in soil moisture^13^ that facilitated biological activity and thus efflux of soil C.

In contrast to drought, experimental moderate warming did not change aNPP across sites compared to their controls (−7 to +25%, *p* = 0.378, Fig. 1b). Warming was expected to increase aNPP^12^; the observed lack of response of aNPP could be due to the moderate temperature increase obtained with the manipulation technique (see also Supplementary Discussion online). Plant communities are long-term ecological units with limited flexibility and may not change fast enough to allow for a stronger response to environmental change on a short time scale^14^,^15^. Warming decreased Rs by up to 5% (*p* = 0.029, Fig. 1b) compared to the controls across sites, excluding the hydric UK site. Warming was expected to affect Rs in multiple ways depending on how environmental change affects plants and the soil matrix by e.g. inducing water limitation^16^, changing chemistry^17^ or soil properties^13^.

We further investigated whether the observed responses of aNPP and Rs were affected by MAP and MAT. We used the position of each site on the Gaussen index, from xeric sites to hydric sites, to describe site-level climate (Fig. 1). The degree to which drought suppressed aNPP did not change along the aridity gradient (linear regression; *p* = 0.544). The effects of drought on aNPP are superimposed on site-specific interannual variability in precipitation; this could have made any subtle pattern in the responses of aNPP more difficult to detect. In contrast to aNPP, the Rs response changed across the Gaussen gradient, with a tendency of suppressed Rs increasing from xeric to mesic sites (Fig. 1a, linear regression; *p* = 0.131), excluding the hydric UK site. The hydric site is seasonally water logged and a drought-induced decrease in soil moisture is stimulating plant and microbial activity; this causes an opposite Rs response compared to xeric and mesic sites where soil water availability is limiting activity.

We expected warming to increase aNPP^12^ and Rs^18^ from xeric sites to the hydric UK site. Instead, warming did not change mean aNPP across the Gaussen index (linear regression; *p* = 0.342) and had a small, negative effect on Rs (Fig. 1b, linear regression; *p* = 0.036). The magnitude of this warming effect decreased from xeric to mesic sites which is in agreement with recent findings that soil C stocks decrease under warming in high-latitude areas^5^. However, the overall small response of aNPP to warming (although highly variably over time) can be attributed to the overall minimal achieved degree of warming (0.2–0.9 °C). The small degree of warming did prolong the plants growing season by 1–2 weeks^9^,^19^ but a warming induced reduction in soil moisture may has prevented a consistent warming response of aNPP.

**Figure 2:**
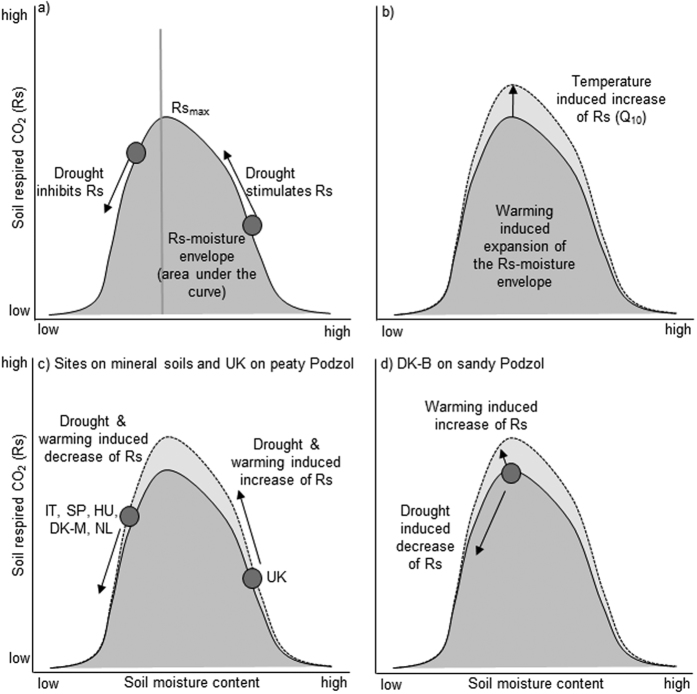
Conceptual framework of the effect of environmental change on the relationship between soil respiration (Rs) and soil moisture. (**a**) The Rs-soil moisture response envelope is shaped by the soil moisture content and other environmental conditions limiting Rs. The shape of the response envelope determines a point where Rs is maximal (Rs_max_). Drought-induced reduction in soil moisture content inhibits Rs up to Rs_max_. Drought stimulates Rs if the soil moisture content is above the soil moisture that stimulates maximum Rs. (**b**) Warming-induced expansion of the Rs-soil moisture response envelope following Q_10_ dynamics. (**c**) Locations of experimental sites on the Rs-soil moisture response envelope concluded from there observed drought and warming effects on Rs and underlying soil types (Table 1). (**d**) Location of the DK-B site on the Rs-soil moisture response envelope. DK-B is the only site that showed a changed Rs response when exposed to drought and warming which is likely mediated by the sandy soil.

The drought and warming responses of Rs compared to the controls were more consistent over time than aNPP responses (Fig. 1; smaller error bars). However, the direction of the Rs response to drought and warming at the hydric UK site differed from the responses at the other sites. We propose that the observed Rs response to environmental change is related to a change in soil moisture^20^ (Fig. 2a). A recent meta-analysis investigated the effect of soil moisture on Rs suggesting that site-specific soil types and plant communities drive observed Rs responses, making predictions difficult^8^. Equations used for modelling the effects of soil moisture on Rs in the meta-analysis follow the general concept that a reduction in soil moisture reduces Rs^8^. However, if soil moisture is naturally so high that it limits Rs due to low soil aeration at the hydric UK site, a reduction in soil moisture will stimulate Rs^20^,^21^. In addition, higher temperatures are expected to increase Rs when soil moisture is constant^4^,^22^ (Fig. 2b).

The differential response of Rs to drought and warming at the non-hydric sites (inhibiting Rs) compared to the hydric UK site (stimulating Rs) can be explained by the location of the sites on an Rs-soil moisture response envelope (Fig. 2c). A soil moisture response envelope is conceptually most representative for the Rs-moisture relationship and helps to explain the observed Rs responses to climate treatments. Reductions in soil moisture under drought will push Rs responses along the response envelope, reducing Rs in xeric and mesic systems but increasing Rs in hydric systems (Fig. 2a).

The DK-B site was the only site where Rs responded differently to both drought and warming compared to the control (Fig. 1, Supplementary Fig. S6). The contrasting response of Rs to drought and warming suggest that the site is positioned at a critical location on the Rs-moisture response envelope (Fig. 2d). The different Rs response of the DK-B site to warming compared to the DK-M site with a similar Gaussen index, similar soil type and plant community may be mediated by the warming induced prolongation of the plant growing season^19^ combined with a very small warming treatment effect (Table 1, DK-B only 0.2 °C). A small warming-induced reduction in soil moisture combined with a significant effect of warming on the plant community can result in Rs being driven by the plant community rather than being limited by soil moisture. Generally, low water holding capacity of soils dominated by coarse particles (DK-B and DK-M ~70% sand) makes Rs more likely to be driven by precipitation events and thus driven by the availability of soil water, as observed at the DK-M site.

We argue that although the use of the Gaussen index, combining MAP and MAT is very useful for demonstrating broad trends of ecosystem responses to climate change, site-specific characteristics like physical properties and soil moisture status are critical to aid understanding as these underpin important processes that are influenced by environmental change. Recent research shows that drought-induced alteration of the soil structure can cause a major shift in soil moisture behaviour^13^ but the impact on Rs remains to be explored.

Moreover, soil water availability is important because it affects plant and microbial processes^16^ that drive terrestrial C turnover. In response to environmental change, plant resource allocation may alter to increase the extent of the root system, thereby maintaining access to resources necessary to sustain growth^22^ and thus ecosystem functioning. However, the observed reductions in Rs under environmental change (Fig. 1) suggest an overall decrease in belowground activity and a decoupling of plant-soil responses of different magnitude across the European climate gradient. The predicted increase in the magnitude and frequency of drought events may lead to a greater Rs response, increasing ecosystem resilience to environmental change in the longer term. However, little evidence of acclimation over the 8–12 years of experimental treatment was observed at our sites.

A change in terrestrial C turnover caused by environmental change or other factors will feed back to the atmosphere^23^. We showed that drought not only decreased aNPP, but also soil C efflux (at least at one hydric site), mitigating potential drought-induced imbalances of the shrubland C budget. Our results suggest that the risk of terrestrial C loss in response to drought is greatest in the order hydric > mesic > xeric systems based on relative change of aNPP and Rs. It remains to be verified that the identified relationship between Rs and soil water status is valid for ecosystems other than shrublands. Dependent on the stresses imposed by natural as well as anthropogenic pressures^1^, ecosystem C dynamics may change considerably and unpredictably^13^ in direction and magnitude. Our long-term climate change manipulation experiments provide insight into the importance of belowground responses, their variability and sensitivity across a climatic gradient in Europe.

## Methods

INCREASE (Integrated Network on Climate Research Activities in Shrubland Ecosystems) is a network of climate change experiments in shrublands spanning a cross-continental gradient in precipitation and temperature^10^. Sites were established in the United Kingdom (UK), The Netherlands (NL), Denmark (DK-B, DK-M), Hungary (HU), Spain (SP) and Italy (IT)^14^. Mean annual precipitation (MAP) and mean annual temperature (MAT) (Table 1) were used to calculate a modified Gaussen index (GI) of aridity for each site as GI = MAP/(2*MAT). MAP and MAT were adjusted for site-specific reductions in MAP induced by the drought treatment and temperature increase via night-time warming (Fig. S2), respectively. Precipitation was excluded by transparent polyethylene plastic curtains that extended over the experimental plots (4m × 5 m). Curtains were activated by precipitation sensors and withdrawn when the rain had stopped. In the warming treatment plots, reflective aluminium curtains, activated by a light sensor at dusk reduced night-time radiative heat losses. Each site had three replicates per treatment, including untreated control plots^10^. Regular measurements of climate variables, standing aboveground plant biomass (aboveground biomass: AGB), litterfall and soil respiration (Rs) were conducted at all sites according to a mutually agreed-upon protocol.

AGB (g biomass m^−2^) was calculated from an annual point intercept survey^14^. A minimum of 300 measurement points were collected at each site along transect lines (IT, SP, HU, NL) or in 0.5 m × 0.5 m square subplots (DK sites, UK). Vegetation plots outside the experimental areas were surveyed and then harvested, and dry weight biomass was used for biomass estimates using point intercept data.

Litterfall (g biomass m^−2^ yr^−1^) was measured using litter collectors. Dependent on the site, 5–30 litter pots (1.5–4.4 cm diameter) were placed randomly beneath the canopy in each plot. At the DK sites, grass litter was estimated based on pin-point measurements. Litter was collected monthly or every 2 or 6 months (site dependent), dried to constant weight at 60 °C and weighed. Where AGB was present for a site but litterfall was not measured separately, average litterfall of all measurements was used instead.

Aboveground net primary production (aNPP, g biomass m^−2^ yr^−1^) is a combination of newly developed photosynthetically active leaves and the (woody) growth increment. aNPP was calculated based on the annual AGB increment and litterfall (IT, SP, DK sites, NL) where the AGB increment is the difference in AGB between following years. If the AGB increment was positive, aNPP was the sum of the increment plus the litterfall of the current year. If the AGB increment was negative then:


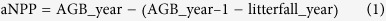


where year was the year of interest and year-1 was the measure of the previous year. If litterfall data was not available for a particular year of AGB measurement, the average litterfall of the site was used instead. aNPP was calculated differently in HU and UK due to the site-specific character of aNPP. In HU, aNPP was similar to the litterfall of the same year as plants were deciduous and shed their leaves before winter. In the UK, aNPP was similar to the litterfall of the previous year as it was the best predictor for aNPP at the site due to the perennial character of the dominant plant *Calluna vulgaris*. AGB (g biomass m^−2^) and aNPP (g biomass m^−2^ yr^−1^) were converted to g C by multiplying by 0.5 (the rough percentage of C content in plant material being 50%).

Rs was measured in three permanent soil collars (10 cm diameter) placed in each plot. All aboveground vegetation was removed from the inside of these collars. Rs was measured biweekly (DK-B) or monthly throughout the year (UK, NL, HU, IT, DK-M) or estimated based on campaign measurements (SP). Rs was up-scaled to annual Rs^9^,^24^,^25^,^26^.

Statistical analyses were performed in R^27^ version 3.0.3. The overall effect of drought and warming on the % change of aNPP and Rs C was tested using students t-test given mu = zero for the control treatment. Temporal site-specific effects of drought and warming on aNPP and Rs over time were characterized using the non-parametric Mann-Kendall test using the R-package “wq” (Supplementary Table S2, Supplementary Figs S4–S10). Effects of drought and warming on mean AGB, aNPP and Rs over the experimental period (Supplementary Table S1, Supplementary Figs S4–S10) were tested using an analysis of variance (ANOVA, all data showed equal variances). Tukey’s HSD test was applied to identify treatment effects. A significance level of 95% was applied where *p-*values below 0.05 show a significant treatment effect on AGB, aNPP or Rs (Supplementary Table S1). The % change of aNPP and Rs induced by the experimentally manipulations was calculated as difference between the treatment and the control treatments. Linear regression was performed to investigate changes in aNPP and Rs across the Gaussen index of aridity.

## Additional Information

**How to cite this article**: Reinsch, S. *et al*. Shrubland primary production and soil respiration diverge along European climate gradient. *Sci. Rep.* **7**, 43952; doi: 10.1038/srep43952 (2017).

**Publisher's note:** Springer Nature remains neutral with regard to jurisdictional claims in published maps and institutional affiliations.

## Supplementary Material

Supplementary Information

## Figures and Tables

**Table 1:** Experimental sites and climate manipulations.

Site Code	UK	NL	DK-B	DK-M	HU	SP	IT
Country	United Kingdom	The Netherlands	Denmark	Denmark	Hungary	Spain	Italy
Site name	Clocaenog	Oldebroek	Brandbjerg	Mols	Kiskunsag	Garraf	Capo Caccia
Coordinates	53°03′N 3°28′W	52°24′N 5°55′E	55°53′N 11°58′E	56°23′N 10°57′E	46°53′N 19°23′E	41°18′N 1°49′E	40°36′N 8°9′E
Start of experiment	1998	1998	2004	1998	2001	1998	2001
First year treatment	1999	1999	2006	1999	2002	1999	2002
MAP (mm)	1263	1005	757	669	558	559	544
MAP red (%)	25	19	8	18	22	49	16
MAT (°C)	7.4	8.9	9.4	8.7	10.5	15.2	16.1
MAT inc (°C)	0.2	0.3	0.2	0.9	0.4	0.6	0.4
Shrubland type	Atlantic heathland	Atlantic heathland	Atlantic heathland	Atlantic heathland	Continental forest steppe	Mediterranean Machia/Garrigue	Mediterranean Machia/Garrigue
Dominant plant species	*Calluna vulgaris*	*Calluna vulgaris*	*Calluna vulgaris, Deschampsia flexuosa*	*Calluna vulgaris, Deschampsia flexuosa*	*Populus alba, Festuca vaginata*	*Erica multiflora, Globularia alypum*	*Cistus monspeliensis, Helichrysum italicum, Dorycnium pentaphyllum*
Soil type (FAO)	Peaty Podzol	Haplic Arenosol	Sandy Podzol	Sandy Podzol	Calcaric Arenosol	Petrocalcic Calcixerept	Luvisol and Leptosol

MAP = mean annual precipitation, MAP red = actual reduction in precipitation (drought treatment), MAT = mean annual temperature, MAT inc = average temperature increase (warming treatment). MAP and MAT for the study periods. Further details are described elsewhere^10^,^14^. A discussion of the effects of the warming treatment on air and soil temperatures can be found in the Supplementary Discussion online.
