# Supplementary material for: Shrubland primary production and soil respiration diverge along European climate gradient
Source: Sci Rep. 2017 Mar 3;7:43952. doi: 10.1038/srep43952 (PMC5335567; doi:10.1038/srep43952)
Supplement: Supplementary Information [file srep43952-s1.pdf]

Supplementary Information

Shrubland primary production and soil respiration diverge along European climate gradient

Sabine Reinsch, Eva Koller, Alwyn Sowerby, Giovanbattista de Dato, Marc Estiarte, Gabriele Guidolotti, Edit Kovács-Láng, György Kröel-Dulay, Eszter Lellei-Kovács, Klaus S. Larsen, Dario Liberati, Josep Peñuelas, Johannes Ransijn, David A. Robinson, Inger K. Schmidt, Andrew R. Smith, Albert Tietema, Jeffrey S. Dukes, Claus Beier, Bridget A. Emmett

Supplementary Figures

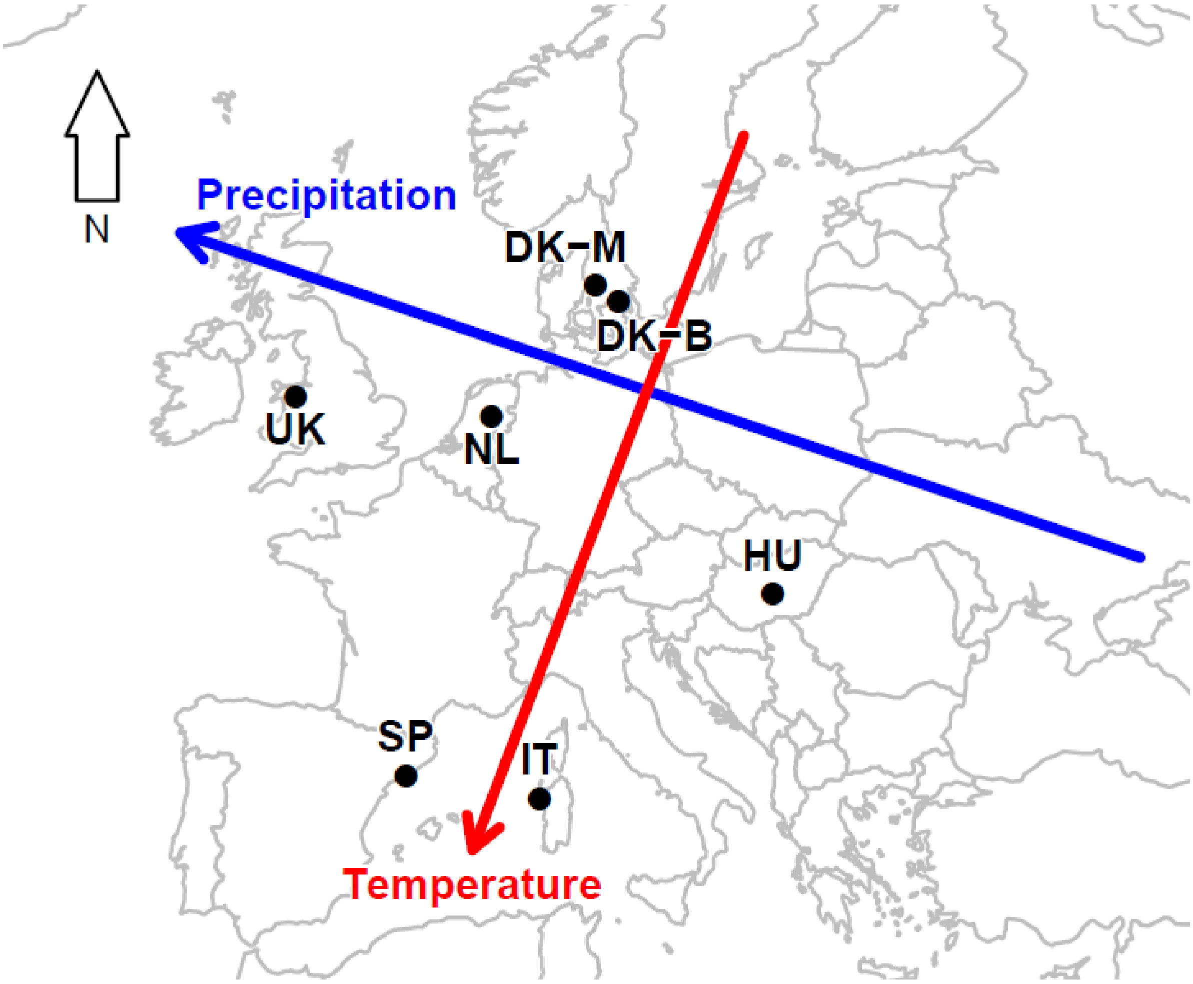

Supplementary Figure S1. Experimental sites across Europe. Sites are located across a precipitation (blue arrow) and temperature gradient (red arrow). IT=Italy, SP=Spain, HU=Hungary, DK-M=Denmark Mols, DK-B=Denmark Brandbjerg, NL=The Netherlands, UK=United Kingdom. The map was created in R[1] using the packages ‘maps’[2], ‘TeachingDemos’[3] and ‘GISTools’[4].

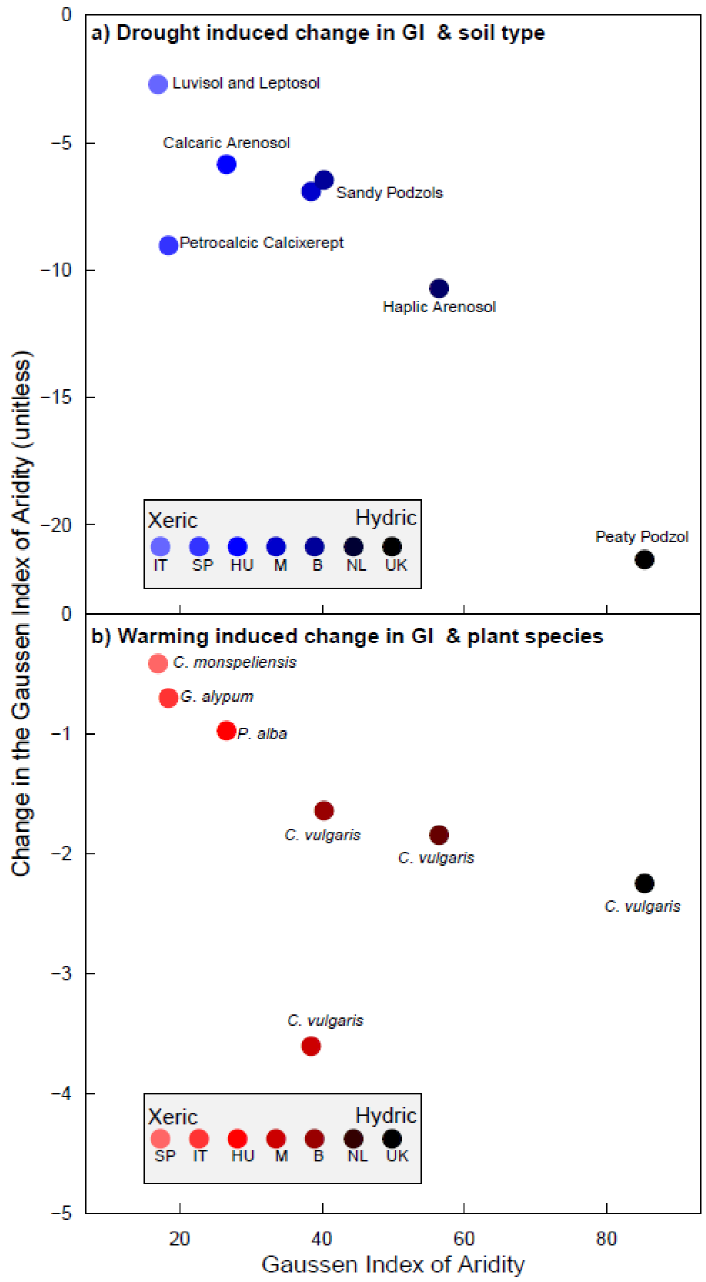

Supplementary Figure S2. Effect of drought and warming on the Gaussen Aridity Index. Change in the Gaussen Index (GI = MAP/2*MAT) from the control (---) due to experimentally induced change in a) precipitation (drought, reduction in MAP) and b) temperature (warming, increase in MAT) for seven European shrublands. The lower the GI, the drier and warmer the site. Soil types and dominant plant species for the sites are detailed in a) and b) respectively. DK-B is co-dominated by *D. flexuosa*, SP is co-dominated by *E. multiflora* (also see Table 1). IT=Italy, SP=Spain, HU=Hungary, M=Denmark Mols, B=Denmark Brandbjerg, NL=The Netherlands, UK=United Kingdom.

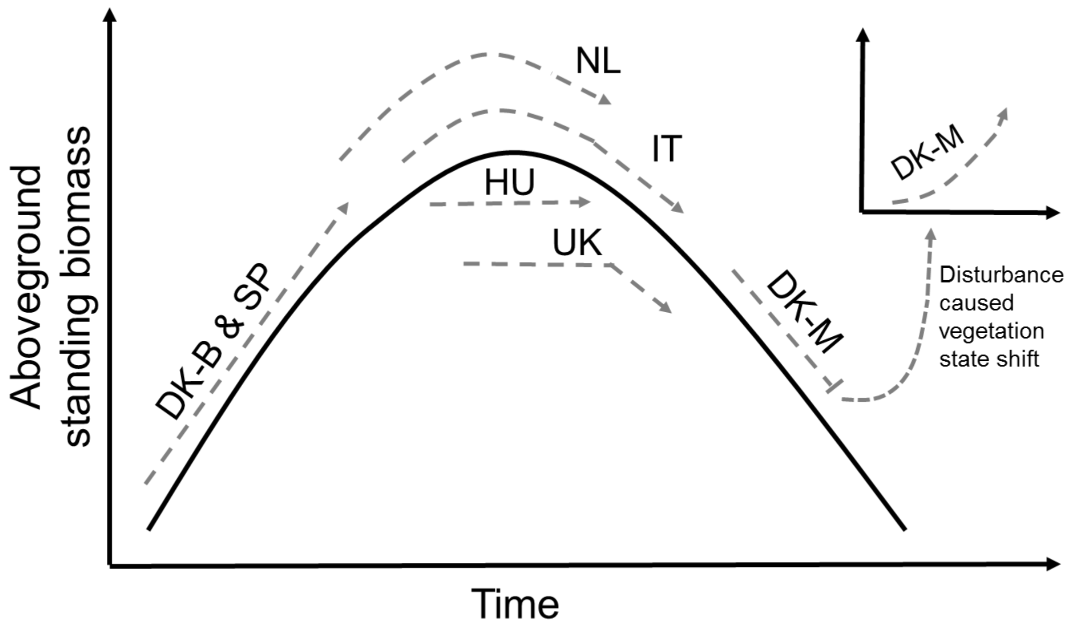

Supplementary Figure S3. States of shrubland biomass development over time. Accumulation of biomass (DK-B, SP), stable or declining biomass (HU, UK), declining biomass after biomass accumulation (IT, NL). The DK-M site experienced a strong heather beetle outbreak that changed the vegetation to the initial state of shrubland development which is dominated by grasses, followed by biomass accumulation. IT=Italy, SP=Spain, HU=Hungary, DK-M=Denmark Mols, DK-B=Denmark Brandbjerg, NL=The Netherlands, UK=United Kingdom.

Supplementary Figures S4-S10. The figures show the long-term pattern of aboveground standing plant biomass (AGB), annual net primary productivity (aNPP) and soil respiration (Rs) for each of the seven experimental sites across Europe. Temporal patterns of the parameters are presented as total values for the untreated control plots and illustrate site specific trends in AGB, aNPP and Rs, respectively. Where applicable, the observed trends in control plots in AGB, aNPP, and Rs are explained in terms of succession and extreme events across the sites and are detailed in the figure captions. The effects of drought and warming on AGB, aNPP and Rs are presented as difference from the control.

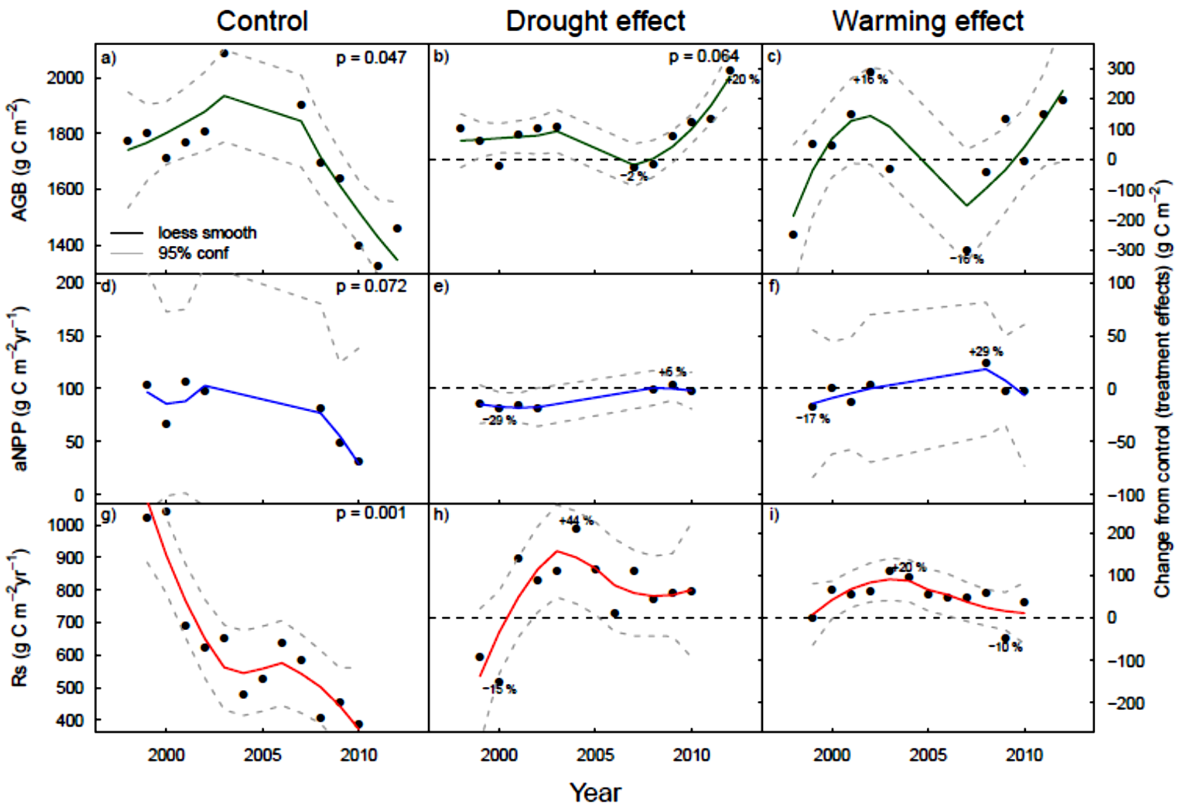

Supplementary Figure S4. Above- and belowground carbon pools and dynamics in the United Kingdom. Aboveground standing plant biomass (AGB, g biomass C m^{-2}), aboveground net primary productivity (aNPP, g C m^{-2} yr^{-1}) and soil respiration (Rs, g C m^{-2} yr^{-1}) over time. (a, d, g) total values with scale on left hand site, (b, e, h) drought effect on measures calculated as difference from control with scale on right hand site, (c, f, i) warming effect on measures calculated as difference from control with scale on right hand site. A loess smooth was applied including a 95 % confidence interval. --- control, values above or below the line = increase or decrease compared to control respectively. Trends over time are analysed using the Mann-Kendall test (Supplementary Table S2). *P-values* are indicated when < 0.1. For drought and warming effects, the most positive and most negative change from the control is given in each panel (in % change).

When the experiment was initiated at the site, the vegetation was a mature heathland with mean AGB C of 1800 g. As a natural course of succession, heathland vegetation dies back slowly (Supplementary Fig. S3), opening up the plant canopy and reducing leaf production[5]. As a consequence, root activity will also decreases. In addition, the experimental site was exposed to a natural extreme summer drought which resulted in soil drying associated with soil C loss which reduced the capacity of the soil to hold water[6]. This change in soil structure and water holding properties affected AGB, aNPP and Rs.

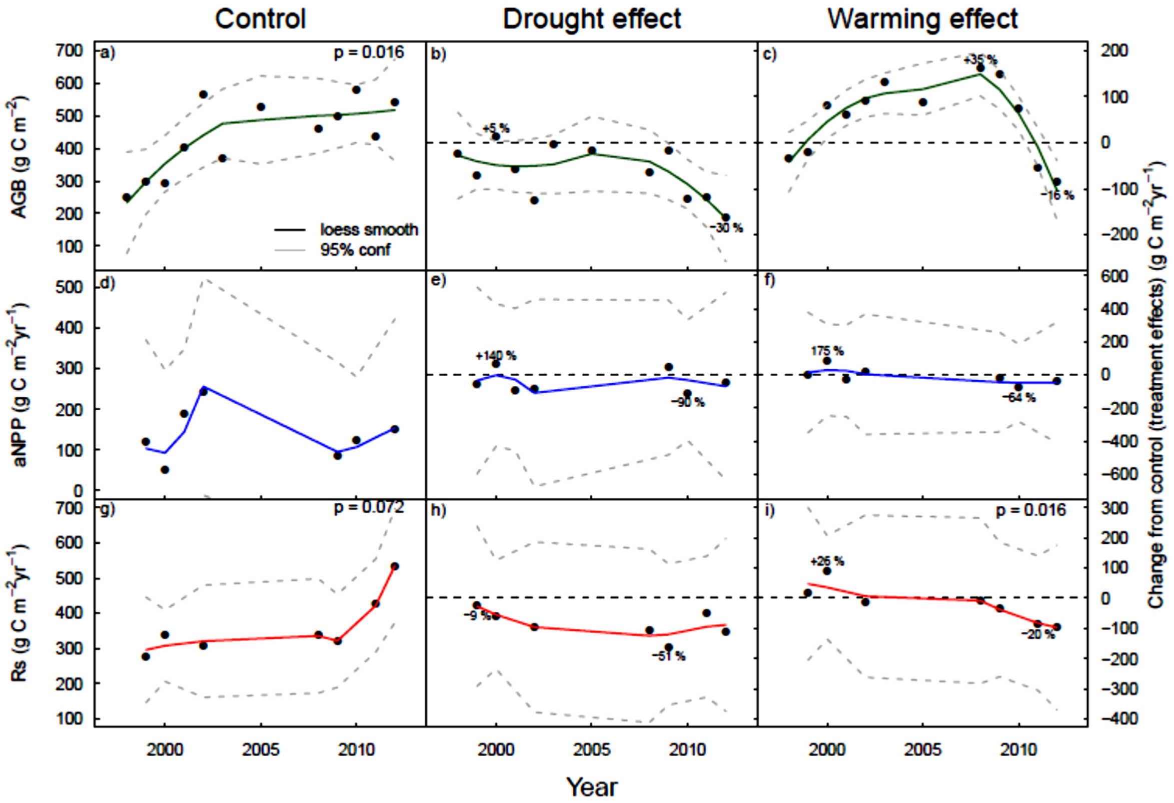

Supplementary Figure S5. Above- and belowground carbon pools and dynamics in the Netherlands. Aboveground standing plant biomass (AGB, g biomass C m^{-2}), aboveground net primary productivity (aNPP, g C m^{-2} yr^{-1}) and soil respiration (Rs, g C m^{-2} yr^{-1}) over time. (a, d, g) total values with scale on left hand site, (b, e, h) drought effect on measures calculated as difference from control with scale on right hand site, (c, f, i) warming effect on measures calculated as difference from control with scale on right hand site. A loess smooth was applied including a 95 % confidence interval. --- control, values above or below the line = increase or decrease compared to control respectively. Trends over time are analysed using the Mann-Kendall test (Supplementary Table S2). *P-values* are indicated when < 0.1. For drought and warming effects, the most positive and most negative change from the control is given in each panel (in % change).

AGB C dynamics show a bell-shaped curve because the plants reach their maximum age (25-30) after this maximum growth phase, plants start to die (Supplementary Fig. S3), gaps develop in the plant canopy, and young *Calluna* plants and bryophytes grow. AGB was very low in 2003 because of a very dry and hot year[7]. Soil respiration increased likely due to more favourable (more stable temperature and moisture) conditions for decomposition of organic matter (increasing heterotrophic respiration) induced by an increased cover of mosses.

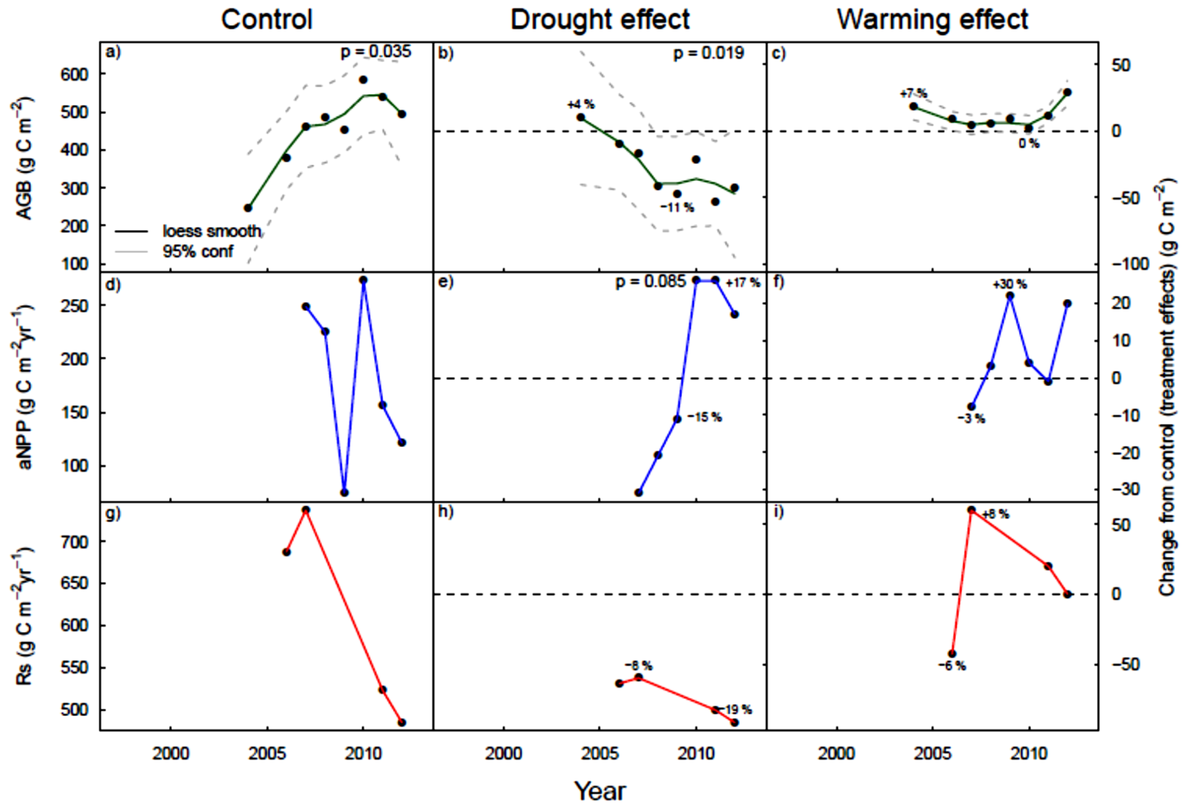

Supplementary Figure S6. Above- and belowground carbon pools and dynamics in Denmark-Brandbjerg. Aboveground standing plant biomass (AGB, g biomass C m^{-2}), aboveground net primary productivity (aNPP, g C m^{-2} yr^{-1}) and soil respiration (Rs, g C m^{-2} yr^{-1}) over time. (a, d, g) total values with scale on left hand site, (b, e, h) drought effect on measures calculated as difference from control with scale on right hand site, (c, f, i) warming effect on measures calculated as difference from control with scale on right hand site. A loess smooth was applied including a 95 % confidence interval if the number of measurements less than 6. --- control, values above or below the line = increase or decrease compared to control respectively. Trends over time are analysed using the Mann-Kendall test (Supplementary Table S2). *P-values* are indicated when < 0.1. For drought and warming effects, the most positive and most negative change from the control is given in each panel (in % change).

AGB tended to increase over the years following a typical pattern of shrubland succession (Supplementary Fig. S3). During the two winters, 2010-11 and 2012-13, the co-dominant *Calluna vulgaris* experienced winter desiccation during periods with sunny weather when soils were still frozen. The frost spells led to a reduction in AGB of *C. vulgaris*. aNPP and Rs followed a similar decreasing pattern in the growing seasons following these events.

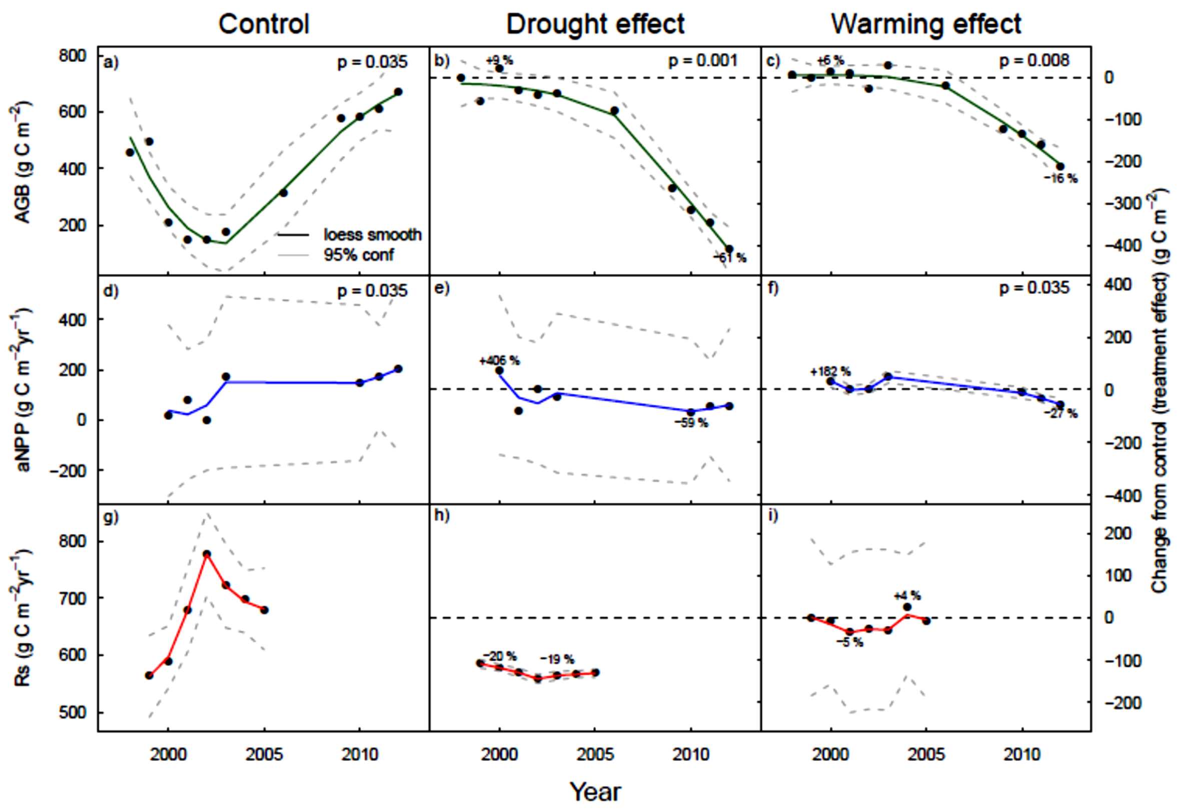

Supplementary Figure S7. Above- and belowground carbon pools and dynamics in Denmark-Mols. Aboveground standing plant biomass (AGB, g biomass C m^{-2}), aboveground net primary productivity (aNPP, g C m^{-2} yr^{-1}) and soil respiration (Rs, g C m^{-2} yr^{-1}) over time. (a, d, g) total values with scale on left hand site, (b, e, h) drought effect on measures calculated as difference from control with scale on right hand site, (c, f, i) warming effect on measures calculated as difference from control with scale on right hand site. A loess smooth was applied including a 95 % confidence interval. --- control, values above or below the line = increase or decrease compared to control respectively. Trends over time are analysed using the Mann-Kendall test (Supplementary Table S2). *P-values* are indicated when < 0.1. For drought and warming effects, the most positive and most negative change from the control is given in each panel (in % change).

Inter annual differences and trends in AGB were largely driven by a major heather beetle outbreak in 1999 and consecutive cutting of the vegetation. After this reduction, biomass tended to increase each year, except from 2009, when a minor heather beetle outbreak occurred. aNPP was reduced due to vegetation loss after the heather beetle outbreak and increased afterwards. Decomposition processes and Rs likely peaked after the disturbance.

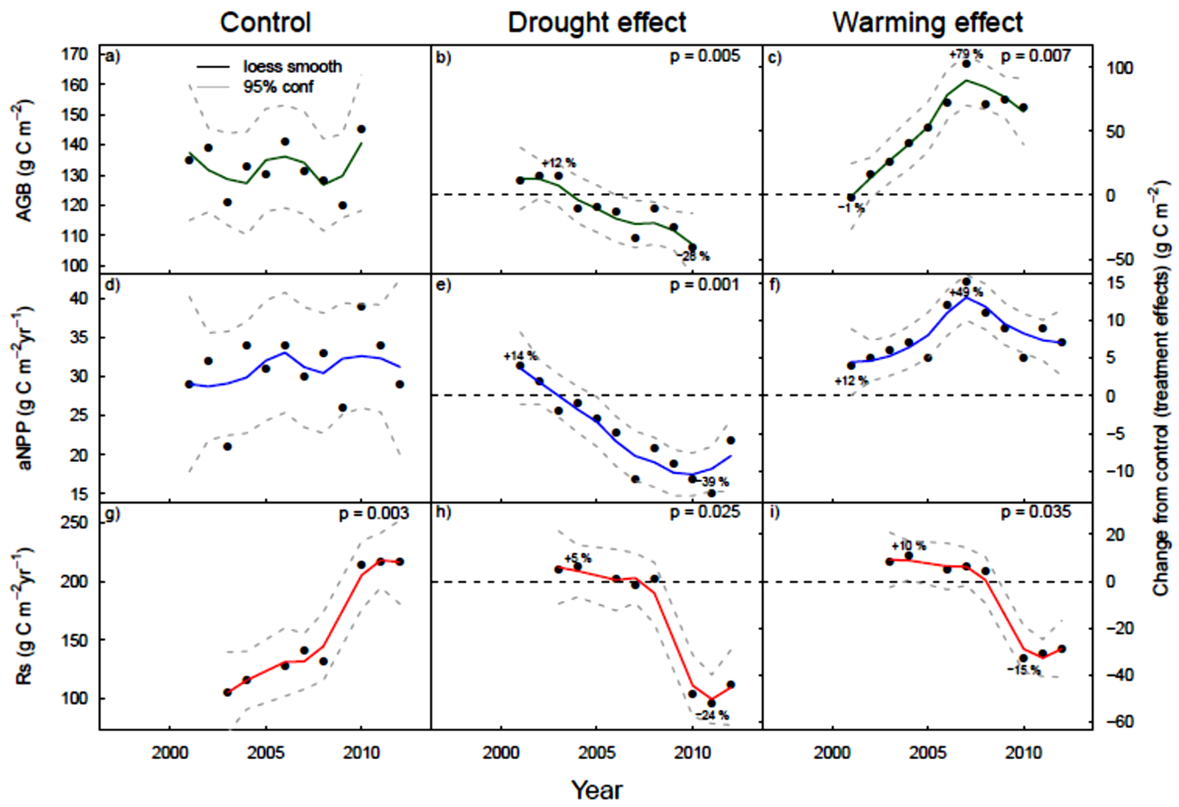

Supplementary Figure S8. Above- and belowground carbon pools and dynamics in Hungary. Aboveground standing plant biomass (AGB, g biomass C m^{-2}), aboveground net primary productivity (aNPP, g C m^{-2} yr^{-1}) and soil respiration (Rs, g C m^{-2} yr^{-1}) over time. (a, d, g) total values with scale on left hand site, (b, e, h) drought effect on measures calculated as difference from control with scale on right hand site, (c, f, i) warming effect on measures calculated as difference from control with scale on right hand site. A loess smooth was applied including a 95 % confidence interval. --- control, values above or below the line = increase or decrease compared to control respectively. Trends over time are analysed using the Mann-Kendall test (Supplementary Table S2). *P-values* are indicated when < 0.1. For drought and warming effects, the most positive and most negative change from the control is given in each panel (in % change).

Inter annual variation in AGB and aNPP was related to variation in precipitation, with 2003 being the driest and 2010 the wettest early summer. The increased Rs in 2010 and afterwards may be related to the increased productivity in 2010, not only aboveground but most likely also belowground. The precipitation-dependency of aNPP has also been shown in Estiarte et *al*. (2016)[8].

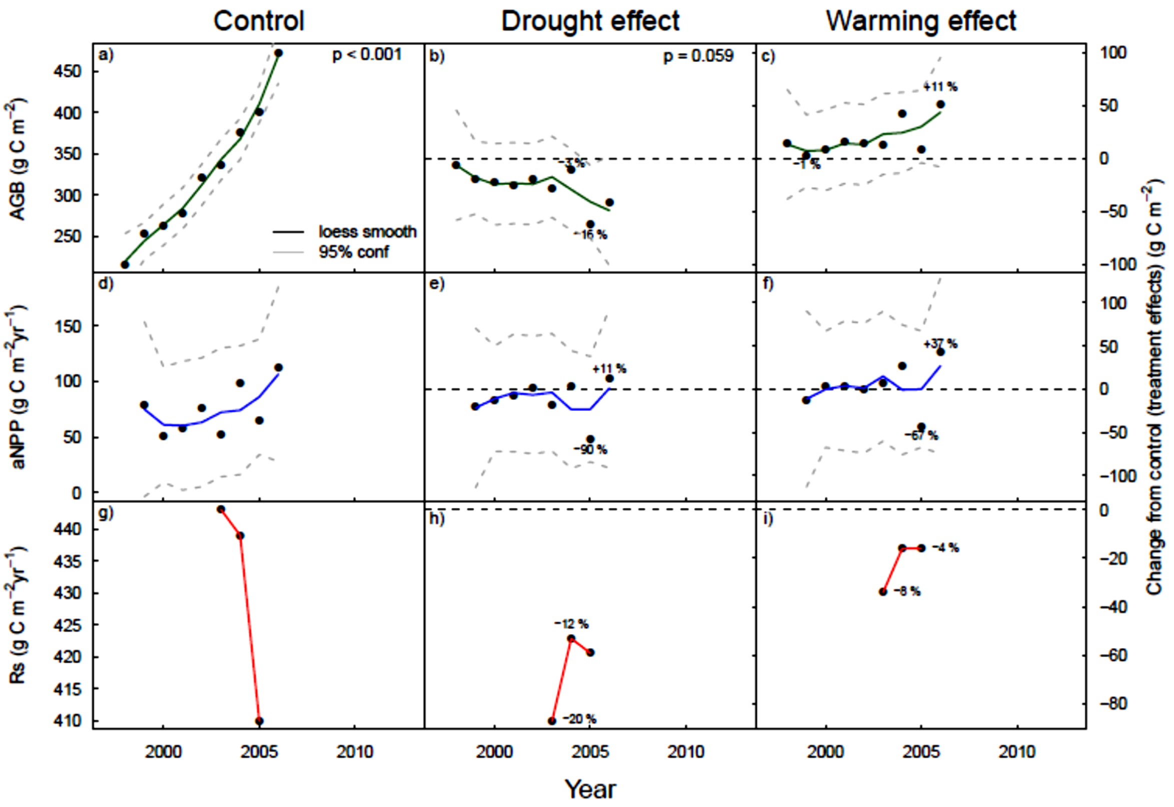

Supplementary Figure S9. Above- and belowground carbon pools and dynamics in Spain. Aboveground standing plant biomass (AGB, g biomass C m^{-2}), aboveground net primary productivity (aNPP, g C m^{-2} yr^{-1}) and soil respiration (Rs, g C m^{-2} yr^{-1}) over time. (a, d, g) total values with scale on left hand site, (b, e, h) drought effect on measures calculated as difference from control with scale on right hand site, (c, f, i) warming effect on measures calculated as difference from control with scale on right hand site. A loess smooth was applied including a 95 % confidence interval if the number of measurements less than 6. --- control, values above or below the line = increase or decrease compared to control respectively. Trends over time are analysed using the Mann-Kendall test (Supplementary Table S2). *P-values* are indicated when < 0.1. For drought and warming effects, the most positive and most negative change from the control is given in each panel (in % change). The progressive increase in AGB is the consequence of the recovery of the vegetation from a forest fire in summer 1994 that removed all the AGB. Main shrub species regrew after the fire leading, in the initial years, to a fast increase in biomass with more than 200 g C m^{-2} in the pre-treatment year 1998. The decrease in Rs in 2005, as compared to the previous two years, was caused by low precipitation in spring.

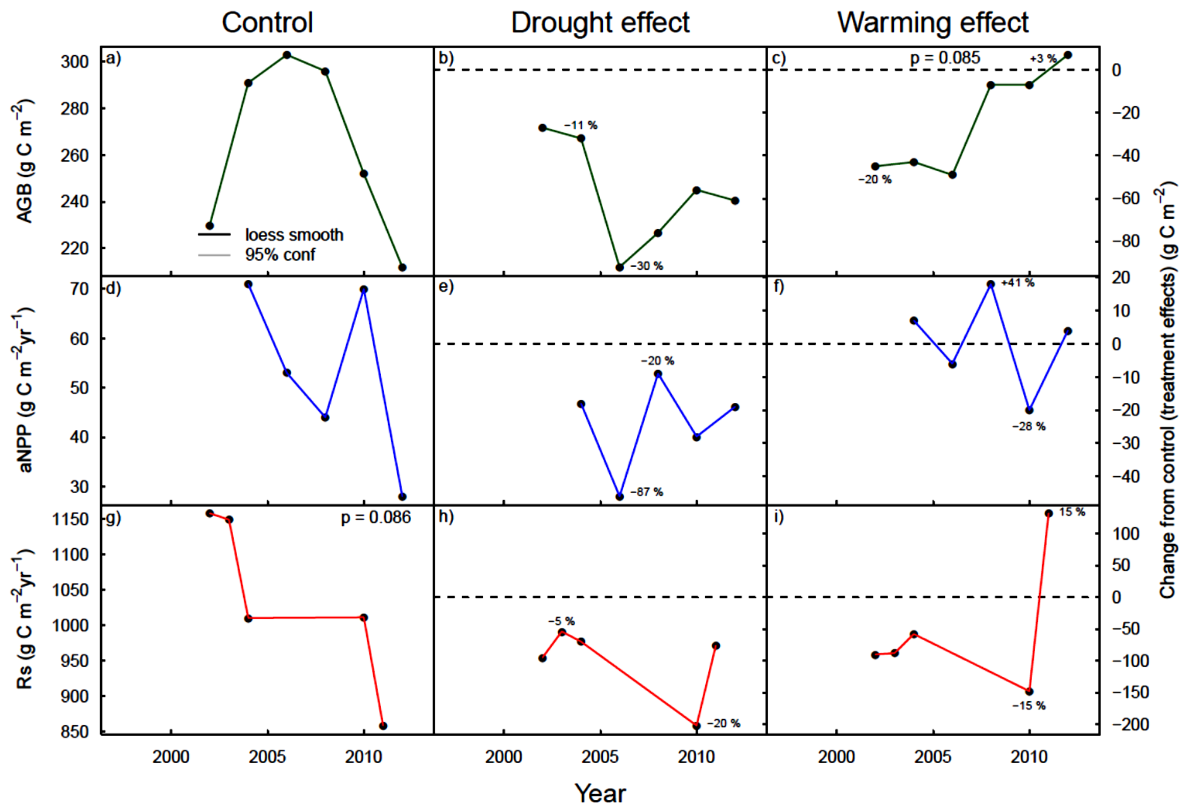

Supplementary Figure S10. Above- and belowground carbon pools and dynamics in Italy. Aboveground standing plant biomass (AGB, g biomass C m^{-2}), aboveground net primary productivity (aNPP, g C m^{-2} yr^{-1}) and soil respiration (Rs, g C m^{-2} yr^{-1}) over time. (a, d, g) total values with scale on left hand site, (b, e, h) drought effect on measures calculated as difference from control with scale on right hand site, (c, f, i) warming effect on measures calculated as difference from control with scale on right hand site. A loess smooth was applied including a 95 % confidence interval if the number of measurements less than 6. --- control, values above or below the line = increase or decrease compared to control respectively. Trends over time are analysed using the Mann-Kendall test (Supplementary Table S2). *P-values* are indicated when < 0.1. For drought and warming effects, the most positive and most negative change from the control is given in each panel (in % change). Changes in AGB C was likely caused by a change in species composition observed over time: in the period 2004-2012, the decline in AGB (occurring despite a slight increase in total plant cover degree) could be related to the reduction of the abundance of *Helichrysum italicum* (in the same period its relative cover degree went from to 18 % to 1 %). The observed decrease in aNPP reflects the general trend observed during succession (Supplementary Fig. S3), with an aNPP increase in the first stages of shrubland formation followed by aNPP decrease in the late stages (the garrigue community was about 20 years old at that time when the experiment was started, and no tree establishment did take place,

which is only occurring in these more recent years after the end of the project). The peak in aNPP observed in 2010 can be connected to the litter production of the senescing plant *H. italicum*, which reached its lowest cover degree in 2010. The decrease in Rs is likely coupled to the decrease in AGB C and thus, overall vegetation activity.

Supplementary Tables

Supplementary Table S1. Mean annual above- and belowground carbon stores. Parameters estimated over the duration of the experiment (Table 1) for each site for N number of years. Analysis of variance was performed to test for differences across treatments within sites with $p < 0.05$ (bold, different letters indicate significant differences) UK=United Kingdom, NL=The Netherlands, DK-M=Denmark-Mols, DK-B=Denmark-Brandbjerg, HU=Hungary, SP=Spain, IT=Italy.

Parameter	Country	N	Control	Drought	Warming
**Standing plant biomass carbon (AGB)** (g C m^{-2} yr^{-1})	UK	12	1698 ± 62	1781 ± 56	1729 ± 64
	NL	8	435 ± 33	371 ± 29	488 ± 44
	DK-M	11	398 ± 62	256 ± 35	340 ± 44
	DK-B	8	455 ± 36	427 ± 31	467 ± 36
	HU	10	**132 ± 3 a**	**122 ± 6 a**	**175 ± 6 b**
	SP	9	324 ± 27	298 ± 24	343 ± 31
	IT	6	264 ± 16	207 ± 14	240 ± 14
**Aboveground net primary productivity (aNPP)** (g C m^{-2} yr^{-1})	UK	7	77 ± 11	67 ± 9	76 ± 11
	NL	8	136 ± 24	83 ± 19	127 ± 27
	DK-M	7	113 ± 30	77 ± 23	111 ± 27
	DK-B	6	183 ± 32	184 ± 33	190 ± 28
	HU	12	**31 ± 1 a**	**26 ± 1 a**	**39 ± 2 b**
	SP	8	74 ± 8	61 ± 14	77 ± 15
	IT	5	53 ± 8	29 ± 9	54 ± 8
**Soil respiration (Rs)** (g C m^{-2} yr^{-1})	UK	12	625 ± 61	684 ± 45	674 ± 62
	NL	7	363 ± 33	276 ± 35	344 ± 24
	DK-M	7	673 ± 28 a	545 ± 23 b	662 ± 26 ab
	DK-B	4	608 ± 62	534 ± 69	618 ± 68
	HU	8	158 ± 17	142 ± 8	151 ± 10
	SP	3	**431 ± 10 a**	**364 ± 11 b**	**409 ± 8 a**
	IT	5	1037 ± 55	938 ± 64	987 ± 38

Supplementary Table S2. Mann-Kendall test as indicator for site specific trends over time. Annual net primary production (aNPP, g C m^{-2} yr^{-1}), standing plant biomass (AGB, g C m^{-2}) and soil respiration (Rs, g C m^{-2} yr^{-1}) were used to test temporal trends for each site. Climate treatments: C=control, D=drought, W=warming, DC=drought effect (D minus C), WC=warming effect (W minus C). The Mann-Kendall-T (Tau) describes the proportion of positive movement over time versus the proportion of negative movement over time looking at all pairwise differences. A *p-value* below 0.05 indicates a significant trend, *p-values* below 0.1 indicate trends. The slope indicates the strength of the direction. UK=United Kingdom, NL=The Netherlands, DK-M=Denmark Mols, DK-B=Denmark Brandbjerg, HU=Hungary, SP=Spain, IT=Italy.

Country	Variable	Stats	C	D	W	DC	WC
		Tau	0.156	-0.388	0.219	-0.748	0.299
	**aNPP**	p-value	0.533	**0.097**	0.367	**0.001**	0.211
		Slope	0.278	-1.000	0.708	-1.600	0.330
		Tau	-0.111	-0.733	0.422	-0.719	0.689
**HU**	**AGB**	p-value	0.721	**0.004**	0.107	**0.005**	**0.007**
		Slope	-0.667	-6.167	3.111	-5.714	9.500
		Tau	0.909	0.857	0.929	-0.691	-0.643
	**Rs**	p-value	**0.003**	**0.004**	**0.002**	**0.025**	**0.035**
		Slope	13.188	6.900	8.361	-5.847	-4.493
		Tau	0.714	0.488	0.524	-0.429	-0.714
	**aNPP**	p-value	**0.035**	0.172	0.133	0.230	**0.035**
		Slope	15.333	7.500	6.000	-6.200	-5.500
		Tau	0.514	-0.018	0.127	-0.782	-0.636
**DK-M**	**AGB**	p-value	**0.035**	1.000	0.640	**0.001**	**0.008**
		Slope	30.000	-1.000	8.000	-29.000	-14.500
		Tau	0.429	0.390	0.524	-0.429	-0.048
	**Rs**	p-value	0.230	0.288	0.133	0.230	1.000
		Slope	21.000	15.600	26.667	-3.667	-0.200
		Tau	-0.333	-0.333	-0.333	0.690	0.333
	**aNPP**	p-value	0.452	0.452	0.452	**0.085**	0.452
		Slope	-24.000	-15.000	-20.000	10.000	4.000
		Tau	0.643	0.571	0.643	-0.714	0.109
**DK-B**	**AGB**	p-value	**0.035**	**0.063**	**0.035**	**0.019**	0.803
		Slope	31.238	23.463	32.225	-7.000	0.988
		tau	-0.667	-0.667	-0.667	-0.667	0.000
	**Rs**	p-value	0.308	0.308	0.308	0.308	1.000
		Slope	-35.917	-42.750	-42.333	-5.208	-1.417
**NL**	**aNPP**	tau	0.238	0.286	-0.230	-0.143	-0.524

		p-value	0.548	0.386	0.548	0.764	0.133
		Slope	3.889	3.788	-4.091	-2.750	-6.000
	**AGB**	tau	0.545	0.382	0.424	-0.333	0.061
		p-value	**0.016**	0.119	**0.064**	0.150	0.837
		Slope	19.883	12.077	21.857	-6.712	2.306
	**Rs**	tau	0.619	0.238	0.238	-0.524	-0.810
		p-value	**0.072**	0.548	0.548	0.133	**0.016**
		Slope	12.250	10.000	3.500	-6.125	-10.667
**UK**	**aNPP**	tau	-0.619	-0.429	-0.143	0.429	0.293
		p-value	**0.072**	0.230	0.764	0.230	0.448
		Slope	-5.400	-3.700	-3.700	1.600	1.375
	**AGB**	tau	-0.455	-0.394	-0.212	0.424	0.242
		p-value	**0.047**	0.086	0.373	**0.064**	0.304
		Slope	-27.101	-16.750	-19.759	5.996	10.615
	**Rs**	tau	-0.727	-0.818	-0.697	0.030	-0.303
		p-value	**0.001**	**0.000**	**0.002**	0.945	0.193
		Slope	-51.586	-41.750	-51.483	1.750	-2.769
**IT**	**aNPP**	tau	-0.600	-0.200	-0.600	0.000	-0.200
		p-value	0.221	0.807	0.221	1.000	0.807
		Slope	-4.333	-3.167	-5.208	1.063	-1.938
	**AGB**	tau	-0.200	-0.467	0.067	-0.333	0.690
		p-value	0.707	0.260	1.000	0.452	**0.085**
		Slope	-3.500	-10.000	3.000	-3.400	6.000
	**Rs**	tau	-0.800	-0.800	-0.600	-0.200	0.400
		p-value	**0.086**	**0.086**	0.221	0.807	0.462
		Slope	-27.524	-31.486	-11.854	-1.804	20.333
**SP**	**aNPP**	tau	0.357	0.143	0.214	0.357	0.400
		p-value	0.266	0.711	0.536	0.266	0.212
		Slope	5.679	9.157	8.750	4.000	5.917
	**AGB**	tau	1.000	0.944	0.944	-0.535	0.343
		p-value	**0.000**	**0.001**	**0.001**	**0.059**	0.246
		Slope	27.550	23.500	28.275	-3.393	3.750
	**Rs**	tau	NA	NA	NA	NA	NA
		p-value	NA	NA	NA	NA	NA
		Slope	-16.500	-2.000	-7.500	14.000	9.000

Supplementary Discussion

Differences in drought and warming treatment effects across sites.

The manipulation of precipitation and temperature were achieved using moveable curtains that removed precipitation (drought treatment) and reduced night-time heat loss (warming treatment)[9]. The drought treatment was imposed during the plants growing season and the duration of the drought corresponded to the last extreme drought event observed at each site prior to the treatment initiation. Thus, timing and magnitude of the drought treatment varied across the sites (Table 1). The warming treatment mimicked the increase in minimum temperatures reported at the time when the experiment was initiated. The retractable curtains covered the plots during night-time and were withdrawn during rain events. At sites with higher precipitation (such as UK and NL), curtains were withdrawn more often, resulting in an overall lower warming effect compared to sites receiving less precipitation (Southern European sites).

Warming treatment and the effect on air and soil temperatures.

The reporting of (average) long-term soil temperature measurements across the seven sites is difficult due to the high degree in variability in soil temperature between sensors depending on location (e.g. depending on canopy cover which could result in several degrees variability across the plot) and the need to remove and reinstall these sensors regularly for calibration. In addition, especially during the early stages of the climate change network (in 1999), temperature sensors were less reliable due to technical problems when the sensors were left in the field and exposed to the various environmental conditions (heat, snow, animals such as mice or rabbits). Air temperature and related meteorological data provide a reliable long-term indicator for the imposed warming effect and for comparability across the European sites in this paper, we report on mean annual air temperatures only (Table 1) as these are the most accurate and comparable measures of the warming treatment that is available. However, the warming methodology has been thoroughly tested within the climate change network and it has been shown that an increase in air temperature as induced by passive night-time warming, resulted in an increase in soil temperature (Supplementary Fig, S11 below)[9,10].

The warming method has been tested at the start of the experiment in 1999 for the UK, DK-M, NL and SP sites and has been reported in Beier et *al*. (2004)[9]. The authors report that an increase in air temperature of ~1.2 ^{o}C as achieved by passive night-time warming (during the night) resulted in an increase in soil temperature of ~1 ^{o}C at 10 cm soil depth but this was highly variable between sites. This is due to several factors including:

1) The variability in the frequency the warming curtains have to be removed during the night to allow rainfall to enter the plots to avoid a drying effect which effectively resulted in only small warming effects or even cooling of the soil. Withdrawal of the curtains was most frequent at the UK site that is exposed to the highest rainfall.
2) The variability in density of canopy cover providing insulation to the soil (highest in NL and UK and least in SP and IT).
3) Dew formation under the curtains from advected air which resulted in a reduction in the warming effect of the soil due to heat evaporating the extra water deposited under the roofs during some seasons (seen most clearly in SP).

This variability in air and soil warming across sites emphasizes the sensitivity of ecosystem processes to warming at the UK site despite having the most modest of soil temperature changes and does not negate our overall findings.

Furthermore, Bruhn et *al*. (2013)[10] critically tested the method of passive night-time warming at the DK-B site using conventional infrared reflective materials as well as insulated curtains to trap more heat underneath the curtains. The authors show that there is a temperature ‘carry over’ from night to day of about 0.5 ^{o}C which is also reflected in the measured soil temperature values which conforms to our observations (Supplementary Fig. S11).

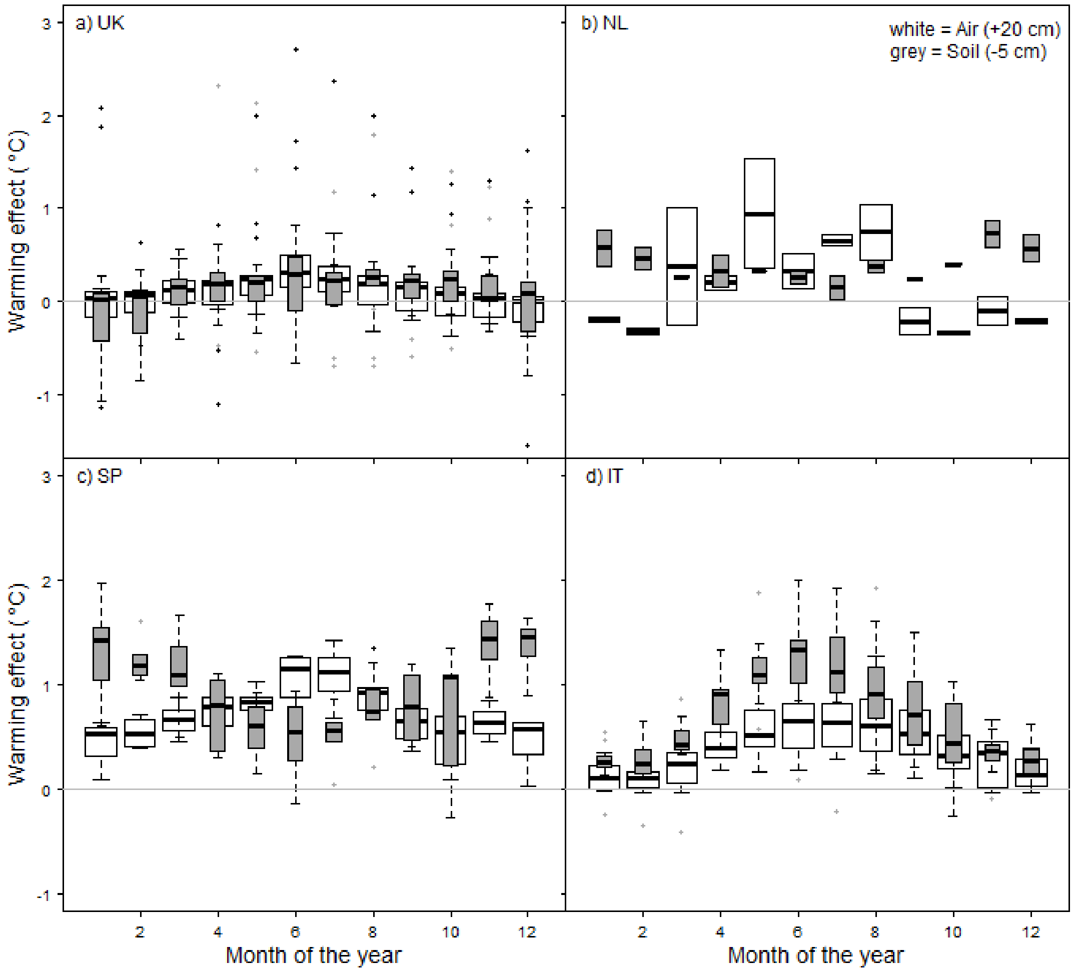

Supplementary Figure 11. Warming effect on air and soil temperatures for two Northern and two Southern European sites. Monthly mean temperatures were calculated as average from the three controls. Average daily temperatures for each treatment were used to calculate monthly averages. The warming effect was calculated by subtraction of the measured temperature in the control treatment from the temperature measured in the warming treatment for air and soil temperatures, respectively. A warming effect of zero (grey line) means that air or soil temperatures were not different in the warming treatment compared to control treatment. a) In the United Kingdom (UK), a small but consistent effect of the warming treatment on air and soil temperatures was observed. Temperature increases occurred mainly during summer months (data: 2000-2006 and 2009-2015), b) In the Netherlands (NL), the warming treatment increased air temperatures during the growing season and soil temperatures were increased throughout the year (data: Sep 2011- Oct 2013), c) In Spain (SP), air temperatures were increased in the summer months whereas soil

temperatures were increased during colder months (data: 2001-2006), d) In Italy (IT), the warming treatment increased air and soil temperatures, respectively (data: 2002-2012).

References

1. R_Core_Team. R: A language and environment for statistical computing. R Foundation for Statistical Computing, Vienna, Austria. URL https://www.R-project.org/. (2016).
2. 'Original S code by Richard A. Becker and Allan R. Wilks. R version by Ray Brownrigg. Enhancements by Thomas P Minka and Alex Deckmyn'. maps: Draw Geographical Maps. R package version 2.3-11. https://CRAN.R-project.org/package=maps. (2015).
3. Snow, G. TeachingDemos: Demonstrations for teaching and learning. R package version 2.9. https://CRAN.R-project.org/package=TeachingDemos. (2013).
4. Brunsdon, C. & Chen, H. GISTools: Some further GIS capabilities for R. R package version 0.7-4. https://CRAN.R-project.org/package=GISTools. (2014).
5. Gimingham, C. H. *Ecology of heathlands*. (Chapman and Hall Ltd, 1972).
6. Robinson, D. A. *et al.* Experimental evidence for drought induced alternative stables states of soil moisture. *Sci. Reports* **6,** 20018 (2016).
7. Ciais, P. *et al.* Europe-wide reduction in primary productivity caused by the heat and drought in 2003. *Nature* **437,** 529–533 (2005).
8. Estiarte, M. *et al.* Few multiyear precipitation-reduction experiments find a shift in the productivity-precipitation relationship. *Glob. Chang. Biol.* **22,** 2570–2581 (2016).
9. Beier, C. *et al.* Novel approaches to study climate change effects on terrestrial ecosystems in the field: Drought and passive nighttime warming. *Ecosystems* **7,** 583–597 (2004).
10. Bruhn, D. *et al.* Improving the performance of infrared reflective night curtains for warming field plots. *Agric. For. Meteorol.* **173,** 53–62 (2013).
